# The Image Behind the Algorithm

**DOI:** 10.1055/s-0046-1825554

**Published:** 2026-07-22

**Authors:** Reinhilde Jacobs, Rellyca Sola Gracea, Dhanaporn Papasratorn

**Affiliations:** 1 OMFS-IMPATH Research Group, Department of Imaging and Pathology, Faculty of Medicine, KU Leuven, Leuven, Belgium; 2 Department of Oral and Maxillofacial Surgery, University Hospitals Leuven, Leuven, Belgium; 3 Department of Dental Medicine, Karolinska Institute, Stockholm, Sweden; 4 Department of Dentomaxillofacial Radiology, Faculty of Dentistry, Universitas Gadjah Mada, Yogyakarta, Indonesia; 5 Department of Oral and Maxillofacial Radiology, Faculty of Dentistry, Mahidol University, Bangkok, Thailand

The dental workforce has never been sufficient to meet the full scope of global oral health needs. The World Health Organization has acknowledged this as a persistent concern, with roughly 3.3 dentists per 10,000 people worldwide, a ratio that masks even sharper disparities between regions and income settings. 1 Oral healthcare workers carry a considerable workload, and are expected to deliver timely, accurate, and high-quality care within an increasingly demanding clinical reality. In this context, artificial intelligence (AI) has begun to attract growing attention in dentistry as a means of supporting clinicians, reducing repetitive cognitive burden, and making better use of the available chair time. 2

Many of the AI models currently being developed for dental practice are trained on data derived from imaging. Over the past decade, AI applications have been developed and are exponentially growing across nearly every dental specialty—from oral pathology detection and periodontal assessment to orthodontic analysis and implant planning, drawing on two-dimensional (2D) modalities such as periapical radiographs, panoramic radiographs, and cephalograms, as well as three-dimensional (3D) data from cone beam computed tomography (CBCT) and conventional CT, and increasingly from intraoral and facial surface scans. The diversity of these applications reflects the breadth of what dental imaging can offer. Yet amid the growing enthusiasm surrounding AI development, a more fundamental question deserves closer attention: not only what these systems can do but also what these AI tools are built upon.

## Not All Images Are Equal

Imaging in dentistry encompasses a wide range of modalities, each with distinct clinical purposes, acquisition characteristics, and diagnostic scope. The contribution that AI can make differs considerably across imaging tasks. The quality, representativeness, and clinical relevance of the underlying imaging data will ultimately determine whether the resulting system is of genuine clinical value or merely of academic interest.

Among the most widely used modalities in daily practice, the intraoral radiograph is also the one where the role of AI warrants careful scrutiny. While offering high spatial resolution of individual teeth and their supporting structures, periapical radiographs are acquired and assessed within seconds, and the added value of AI must be judged against the risk of distracting from, or even obscuring, subtle diagnostic details. Extensive annotations and segmentation masks, detection boxes, or visual overlays may cover precisely the fine structures that make intraoral radiographs clinically valuable. This does not preclude meaningful AI applications in intraoral imaging, but it does require that these AI tools be carefully designed, minimally intrusive, and demonstrably useful for the task at hand following an intense period of training, testing, and clinical validation based on high-end professional expertise. Scientific validation should preferably be present. Unfortunately, at this moment, these prerequisites are rarely met.

The panoramic radiograph presents a different picture. Obtained in less than a minute, a single image encompasses the full dentition, alveolar bone, temporomandibular joints, maxillary sinuses, and a range of anatomical structures whose thorough assessment requires considerably more time and a level of consistency that is difficult to maintain across a full clinical day. It is here that AI can offer something genuinely meaningful, reducing interobserver variability, supporting systematic review, and flagging findings that might otherwise be overlooked ( Fig. 1 ). 3

**Fig. 1:**
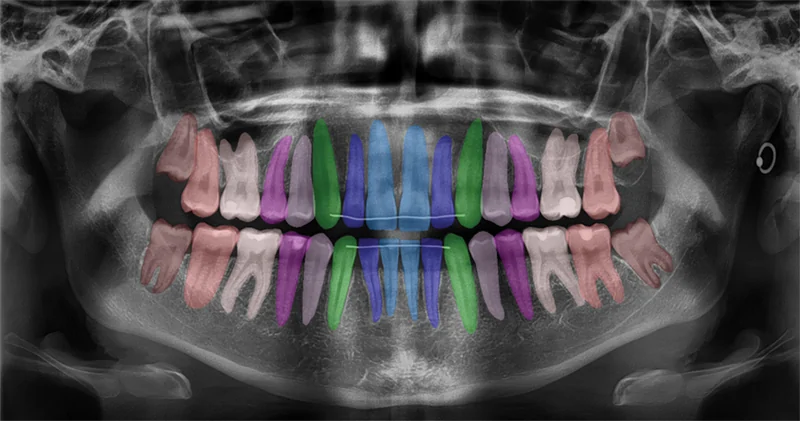
Accurate and efficient AI-based tooth detection and segmentation on panoramic radiographs, by prior highly qualitative human expert training. 3

The transition from 2D to 3D imaging carries further clinical implications. CBCT has expanded what is visible in dental and maxillofacial diagnosis, providing spatial information that 2D imaging alone cannot offer. It allows assessment of bone volume, root morphology, airway anatomy, and pathological extent in three dimensions, and has become indispensable in specific contexts such as implant planning, orthognathic surgery, and assessment of complex pathology. CBCT diagnostic interpretation and treatment planning require specialized training, and thus, for any AI-based CBCT training, supervised learning by highly qualified experts is mandatory. By supporting segmentation, landmark identification, and structured reporting, AI tools may assist clinicians in efficiently extracting more consistent diagnostic information from volumetric datasets ( Fig. 2 ). At the same time, multimodal integration may support treatment planning and longitudinal follow-up. 4 5 If imaging is the clinical substrate of dental AI, then the next challenge lies in how these images are selected, annotated, shared, and governed to optimally support diagnosis and treatment planning.

**Fig. 2:**
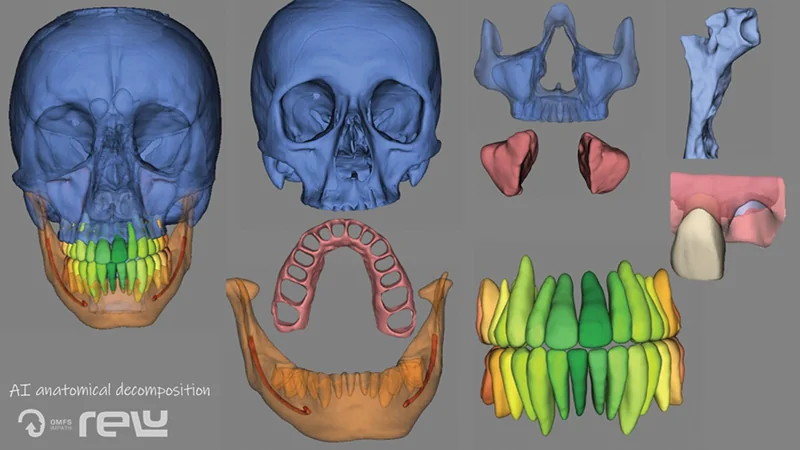
Accurate and efficient AI-based anatomical segmentation with (de)composition of hard and soft tissues as a basis of a 3D virtual patient creator for 3D-based treatment planning. 4

## Trusting Humans and Images Behind AI

High-quality data are the foundation of any reliable AI system. In the context of dental imaging, this means having well-annotated radiographs that accurately reflect clinical reality. When annotations are precise, AI models can learn effectively and provide more accurate predictions for new cases. However, creating such datasets requires considerable time, expertise, and resources. Daily practice involves significant variation, including differences in patient populations, imaging devices, and acquisition settings, all of which affect how images appear. A robust AI model must learn to handle this variability, which requires large, diverse, and carefully curated datasets.

At the same time, patient privacy remains a major limitation. Sharing radiographic data across institutions or countries is often restricted. Federated learning has been proposed as a solution. Rather than sharing images, institutions can train models locally and share only the learned parameters (“learning experience” of the model), which are then combined into a global model. In principle, this enables collaboration without the need to transfer sensitive patient data, and previous studies have shown that it can outperform models trained on single-center datasets.

However, this approach also raises important questions. If the data are never shared, how can we fully understand what the model has learned? Might the shared parameters still reflect local biases, imaging artefacts, or annotation inconsistencies from individual centers? Given the variability in interpreting radiographs, there is a risk that such biases may be incorporated into the final model without being easily detected. This underlines the importance of consistent labelling protocols, quality control, and transparent development processes.

It is also worth asking whether a single global model is always the best objective. In some cases, models adapted to local populations and imaging protocols and validated locally may be more appropriate.

Another development is the increasing use of synthetic images to support AI training. Techniques such as generative adversarial networks (GANs) train two models together to create realistic medical images: one generates images, while the other evaluates their realism. These methods have been employed for data augmentation, noise reduction, and the generation of new images that resemble real clinical cases. Interestingly, some studies report that even experienced clinicians cannot reliably distinguish synthetic images from real ones. Furthermore, the field has evolved beyond GANs, with diffusion-based generative models emerging as a compelling alternative capable of producing synthetic images of comparable or superior quality.

This capability offers clear benefits. Synthetic data can address the issue of limited datasets, particularly for rare conditions, and support research and education by enhancing the learning experience through a variety of cases. However, it also raises important considerations. The same underlying technology is used in “deepfake” systems, which create highly realistic yet artificial images. In a clinical context, an image's realism does not guarantee its accuracy in representing the true disease.

Another concern is what happens when synthetic data are reused repeatedly. If AI-generated images are included in training datasets and future models are then trained on a mixture of real and synthetic data, deviations from true clinical patterns may accumulate over time. This could make it increasingly difficult to determine whether the model still reflects real biological variation—a phenomenon sometimes referred to as “model drift” or “collapse.”

However, these developments do not diminish the potential value of AI in oral healthcare. Rather, they highlight the need for careful implementation. As AI systems become more integrated into clinical workflows, key questions remain: How can we ensure that models are trained on data that accurately represent patients? How transparent are these systems to clinicians? And how much should we rely on outputs that we cannot verify directly?

Trust in AI will not be earned through performance metrics alone, but through the quality of the clinical image data and human expert annotation and validation behind the AI. As AI becomes more deeply embedded in digital dental workflows, the responsibility to ensure that these systems are clinically sound, developed transparently, and rigorously validated becomes shared, extending to researchers, clinicians, educators, and developers. Imaging has always been key to diagnostic oral healthcare. For AI to truly support clinicians in delivering high-quality, efficient oral healthcare, dental AI tools must be developed with the same rigor as clinical expert decision-making.
